# Antimicrobial and Antibiofilm Effects of Peptides from Venom of Social Wasp and Scorpion on Multidrug-Resistant *Acinetobacter baumannii*

**DOI:** 10.3390/toxins11040216

**Published:** 2019-04-10

**Authors:** Rogério Coutinho das Neves, Márcia Renata Mortari, Elisabeth Ferroni Schwartz, André Kipnis, Ana Paula Junqueira-Kipnis

**Affiliations:** 1 Laboratory of Immunopathology of infectious diseases, Department of Immunology, Institute of Tropical Pathology and Public Health, Federal University of Goiás, Rua 235, Goiania, 74605-050 Goiás, Brazil; rogeriocdasneves@hotmail.com (R.C.d.N.); andre.kipnis@gmail.com (A.K.); 2 Laboratory of Neuropharmacology, Department of Physiological Sciences, Institute of Biological Sciences, University of Brasília, 70910-900 Brasilia, Brazil; mamortari@gmail.com (M.R.M.); beth.ferroni@gmail.com (E.F.S.)

**Keywords:** AMP, mastoparan, *Acinetobacter baumannii*, stent

## Abstract

Intravascular stent infection is a rare complication with a high morbidity and high mortality; bacteria from the hospital environment form biofilms and are often multidrug-resistant (MDR). Antimicrobial peptides (AMPs) have been considered as alternatives to bacterial infection treatment. We analyzed the formation of the bacterial biofilm on the vascular stents and also tested the inhibition of this biofilm by AMPs to be used as treatment or coating. Antimicrobial activity and antibiofilm were tested with wasp (Agelaia-MPI, Polybia-MPII, Polydim-I) and scorpion (Con10 and NDBP5.8) AMPs against *Acinetobacter baumannii* clinical strains. *A. baumannii* formed a biofilm on the vascular stent. Agelaia-MPI and Polybia-MPII inhibited biofilm formation with bacterial cell wall degradation. Coating biofilms with polyethylene glycol (PEG 400) and Agelaia-MPI reduced 90% of *A. baumannii* adhesion on stents. The wasp AMPs Agelaia-MPI and Polybia-MPII had better action against MDR *A. baumannii* adherence and biofilm formation on vascular stents, preventing its formation and treating mature biofilm when compared to the other tested peptides.

## 1. Introduction

The use of synthetic materials, such as ureter catheters and urinary stents for temporary or permanent insertion in the body may result in bacterial infections associated with colonization, which is important in the cases of morbidity and can lead to systemic dissemination [1,2]. Treatment with conventional antibiotics against bacterial biofilms formed on implants is inefficient to eradicate the infecting microorganism due to its low bacterial metabolic activity and biofilm protective matrix [3], resulting in a chronic infection of difficult treatment that requires the implant to be removed. Cases of vascular stent infections are rare complications, but associated with high mortality rates; according to current data, mortality may reach 40%, despite antibiotic treatment and/or surgical removal [4,5]. The most likely cause of stent infections is equipment reuse, such as balloons, catheters, and guide-wire, or poor ascetical techniques during the procedure [6]. These bacteria from the hospital environment and human skin are the most frequently found in stent infections: *Staphylococcus* spp. [6,7,8], *Streptococcus* spp. [9], *Pseudomonas* spp. [10,11], Fungi [12], and, in rare cases, rapidly growing mycobacteria (RGM) have also been reported [13]. Different case reports have been published showing cases of patients with stent infection, however, the relationship between the implantation of vascular stent with the development of nosocomial infection and the formation of bacterial biofilm is still not clear. Bacterial adhesion on the surface of the vascular stent material as well as the formation of bacterial biofilm has not been studied. 

Vascular stents are used to increase the luminal diameter of the coronary arteries. The use of drugs to coat stents drastically reduces the process of re-stenosis [14,15]. Its expandable metal composition, whether coated with any drug or not, has a structure comprising a metal core of cobalt–chromium alloy or stainless steel and on the outside may have a coating of two polymers, lactic acid-co-glycolic acid (PLGA) or polylactic acid (PLA) that, in turn, can be manipulated to have additional drugs or antibodies integrated [16,17,18]. Some of the most commonly used drugs in stents are immunosuppressants, such as sirulimus, to reduce the risk of stent thrombosis caused by cell rejection [17]. In addition to implant intervention, patients still use anticoagulants or antiplatelet agents, which prevents the formation of thrombi on the stent [19].

The use of drug-eluting stents (DES) has increased recently, in comparison to the use of bare metal stents, however, DES have been shown to be more susceptible to infections [20]. Stent implantation may result in inflammation that could favor the formation of a conditioning film such as that shown for ureter stents [21]. This conditioning film facilitates bacterial adhesion and biofilm formation [22]. The metallic structure of the stent acts as a nest for bacterial colonization, increasing the risk of dissemination to the arterial wall, causing inflammation, necrosis, and ultimately vessel rupture [23]. Together with the fact that stents are implanted in a hospital setting, with a high prevalence of multidrug-resistant (MDR) bacteria, the risk of biofilm formation by MDR bacteria on biofilm poses an additional realistic threat. 

One of the main bacteria responsible for nosocomial infections is *Acinetobacter baumannii* [24]. This Gram-negative coccobacillus commonly found on skin, in the respiratory tract, and in hospital environments has increased survival rates and the ability to produce biofilm [25,26]. *Acinetobacter* spp. are more frequently found in the intensive care units (ICU) than *Staphylococcus aureus* and *Pseudomonas* spp. [27]. Additionally, 80% of *A. baumannii* clinical isolates were shown to have some type of carbapenem resistance associated with high mortality rates [28]. However, although there are works showing *A. baumannii* biofilm formation in ureters [29] and vascular catheters [30], as well as treatment with different antibiotics against those biofilms, to our knowledge, no study has been done on the ability of *A. baumannii* to form biofilms on cobalt–chromium stents. 

Biofilms made of MDR strains makes the treatment using conventional antibiotics more challenging. New therapeutic alternatives are necessary for these types of cases and antimicrobial peptides (AMP) are promising choices. AMPs are typically less than 100 amino acids in length that exhibit antimicrobial activity and can be obtained from the poisons of various animals, such as wasps [31], ants [32], bees [33], spiders [34], and scorpions [35]. Mastoparan (MP) peptides, which are the most commonly isolated peptide class from the *Vespidae* venom [31], present 10–14 amino acids that include distinct hydrophobic amino acids and an amphipathic helix conformation, which confers broad-spectrum antimicrobial activity against Gram-positive and Gram-negative bacteria [36], fungi [37], and mycobacteria [31,38]. Many AMPs are also present in the scorpion venom, which are classified as AMPs presenting disulfide bridges and AMPs that do not [39]. AMPs have a broad antimicrobial spectrum and are not affected by classical mechanisms of resistance to conventional antibiotics. AMPs interact primarily with the lipids of cytoplasmic membranes or cell walls leading to membrane permeabilization, cell lysis, and death [40]. AMP interaction with the lipid monolayer as described by Brogden (2005) can cause peptide aggregation forming pores, lipid and peptide combination forming a toroidal pore, or direct membrane disruption [41]. This unique mechanism of action allows AMPs to act on bacteria at different biofilm stages such as attachment, structure, and dispersion [42]. 

Therefore, our objectives were to analyze MDR *A. baumannii* biofilm formation on cobalt–chromium coronary stents and to evaluate the action of several antimicrobial peptides from wasp and scorpion venoms against those biofilms.

## 2. Results

### 2.1. Biofilm Formation by A. baumannii Clinical Isolates 

**Figure 1:**
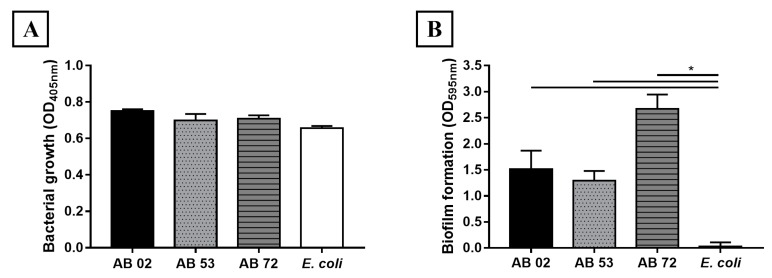
*Acinetobacter baumannii* biofilm formation. (**A**) The bacterial growth of *Acinetobacter baumannii* AB 02, AB 53, AB 72, and *Escherichia coli* (control) were incubated with LB + Glu for 24 h at 29 °C and the growth was determined by OD readings at 405 nm. (**B**) After this period, the presence of biofilms was evaluated using crystal violet dye staining. The bars represent the mean and standard deviations of triplicates. * Significant difference between biofilm formations by *A. baumannii* clinical isolates compared to *E. coli* (*p* < 0.05).

In this work, three *A. baumannii* isolates previously described by Castilho et al. that were isolated from patients with hospital-acquired infections were used [43]. Isolates AB 02 and AB 72 were resistant to ampicillin, amikacin, and ciprofloxacin. AB 53 isolate only presented resistance to ampicillin. All isolates showed intermediate susceptibility to tetracycline, while all isolates were susceptible to meropenem [43]. Thus, AB 02 and AB 72 were considered as MDR strains. The ability to form biofilms by *A. baumannii* isolates AB 02, AB 53 and AB 72 was determined by crystal violet staining of cultures in 96 polystyrene well plates. Figure 1A shows that bacterial growth in the plates were similar between all isolates and *Escherichia coli*, but biofilm formation occurred only with *A. baumannii* isolates (Figure 1B). The AB 72 isolate produced more biofilm than the other isolates. Considering that isolate AB 72 presented resistance to three antimicrobial drugs and showed the highest capacity to form biofilm, we decided to test its ability to adhere to the cobalt chromium vascular stent.

### 2.2. Adhesion and Early Formation of Biofilm on Cobalt–Chromium Vascular Stent

**Figure 2:**
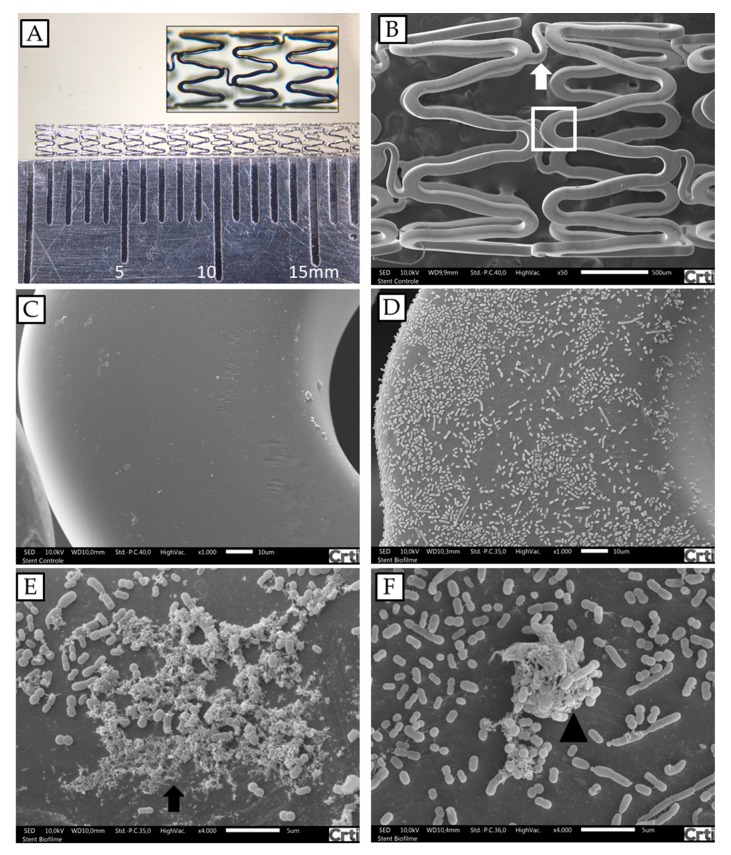
*A. baumannii* biofilm formations on fragments of cobalt–chromium vascular stents analyzed by SEM. (**A**) Coronary stent photography; the fragments used in the experiments were 6 mm long. (**B**) Architecture of the cobalt–chromium vascular stent, presenting two cells, connected by a link (arrow). (**C**) Stent structure enlargement of box in B. (**D** and **E**) Stent after incubation with *A. baumannii* AB 72 for 24 h under conditions for biofilm formation (arrow). (**F**) Protuberant bacterial accumulation, suggestive of initial biofilm formation (arrow head). Magnifications: (**B**) 40×; (**C**) and (**D**) 1000×; (**E**) and (**F**) 4000×.

In order to determine if *A. baumannii* was able to adhere to stents, a cobalt–chromium stent was incubated with AB 72 isolate for 24 h. Figure 2 shows the results of scanning electronic microscopy (SEM) and the colony forming units (CFU) of bacteria recovered from the stents. The first image (Figure 2A) reveals the framework and the configuration of the coronary stent used. Fragments with five cells were used for the analyses. In the SEM analyses (Figure 2C,D) the bacteria adhered to the stent and secreted substances that also adhered to the stent and to the bacterial colonies indicating biofilm formation (Figure 2E). Determination of the bacterial load attached to the stents resulted in 1.3 × 10^6^ CFU per used stent. These results represent one of three independent experiments.

### 2.3. Determination of Minimum Inhibitory Concentration of Antimicrobial Peptides against Isolates of A. baumannii 

Since *A. baumannii* was shown to form biofilm on stents, we first investigated if AMPs derived from arthropod venom were active against these bacteria. Agelaia-MPI, Polybia-MPII, and Polydim-I derived from wasp venom and Con10 or NBDP-5.8 derived from scorpion venom were used. The hydrophobicity evaluation of the peptides showed a range of 0.435 to 0.795 (Con10 < NDBP-5.8 < Polybia-MPII < Agelaia-MPI < Polydim-I) (Table 1). Agelaia-MPI and Polybia-MPII peptides were similar peptides differing by two amino acids. In the 9th position an alanine present in Agelaia-MPI is substituted by a methionine in Polybia-MPII and an isoleucine is substituted by a valine in the 10th position in Polybia-MPII, but their hydrophobicities were maintained (Table 1). 

**Figure 3:**
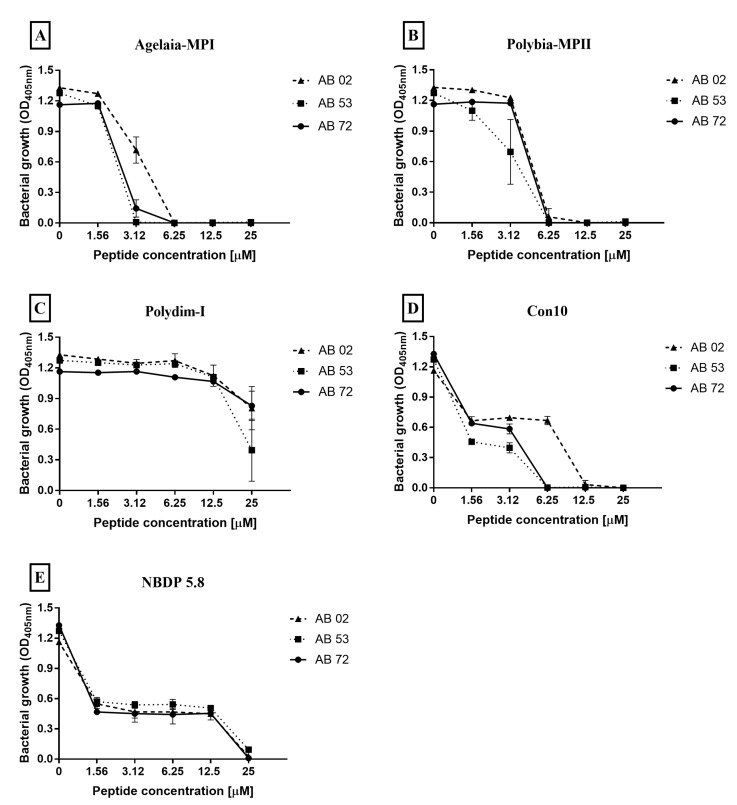
Effect of the antimicrobial peptides (**A**) Agelaia-MPI, (**B**) Polybia-MPII, (**C**) Polydim-I, (**D**) Con10, and (**E**) NBDP 5.8 on the inhibition of adhesion of *A. baumannii* to 96-well polystyrene plates. The curves represent the bacterial growth of adhered cells on polystyrene plate by reading OD in the range of 405 nm. Results are reported as mean and standard deviations of triplicates. These results represent one of three independent experiments.

The ability of AMPs (Agelaia-MPI, Polybia-MPII and Polydim-I, Con10 and NBDP-5.8) to inhibit the bacteria growth by incubating them with three different MDR *A. baumannii* isolates for 24 h was analyzed (Figure 3). The MIC for Agelaia-MPI peptide against AB 02 and AB 72 isolates was 6.25 μM and against AB 53 was 3.12 μM (Figure 3A). Polybia-MPII presented an MIC of 12.5 μM for AB 02 and 6.25 μM for both AB 53 and AB 72 isolates (Figure 3B). Polydim-1 did not completely inhibit the growth of any isolate at the tested concentrations (Figure 3C). The Con10 AMP presented an MIC of 12.5 μM for the AB 02 isolate and 6.25 μM for both AB 53 and AB 72 isolates (Figure 3D). NBDP 5.8 showed a MIC of 25 μM for all isolates analyzed (Figure 3E).

### 2.4. Impact of Antimicrobial Peptides on Bacterial Biofilm Formation 

Since the AMPs were shown to act against *A. baumannii* isolates, we then investigated if they could avoid the formation of biofilm in 96-well plates, calculating the minimum biofilm eradication concentration (MBEC). Agelaia-MPI showed adhesion inhibition for isolates AB 02 at a concentration of 25 μM while isolates AB 53 and AB 72 were inhibited at a concentration of 6.25 and 12.5 μM, respectively (Table 2). Polybia-MPII inhibited at the minimum concentration of 25 μM for AB 02 and AB 72 and 12.5 μM for AB 53 (Table 2). Polydim-I showed low adhesion inhibition—50% for the AB 53 isolate at a concentration greater than 25 μM (Table 2). The Con10 scorpion peptide inhibited the biofilm formation at the concentration of 12.5 μM for the AB 53 and 72 isolates and for the AB 02 isolate the minimum concentration was 25 μM (Table 2). For NBDP 5.8 peptide, it was able to inhibit the biofilm (>95%) of the three isolates at a minimum concentration of 25 μM (Table 2). Therefore, Agelaia-MPI e Polybia-MPII peptides that presented best activities against biofilm formation were selected.

### 2.5. Effect of the Agelaia-MPI and Polybia-MPII Peptides on Mature Biofilm and on the Dispersion of Adherent Cells 

**Figure 4:**
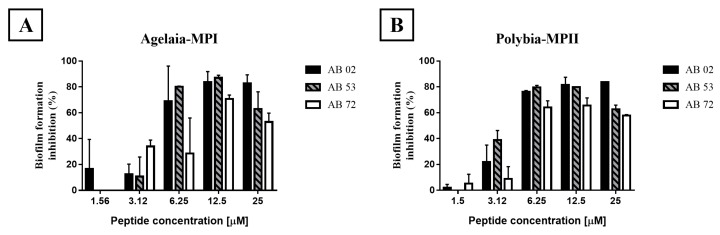
Effect of the antimicrobial peptides Agelaia-MPI and Polybia-MPII on the mature biofilm of *A. baumannii* on polystyrene plates. (**A**) Agelaia-MPI and (**B**) Polybia-MPII significantly inhibited mature biofilm at the lowest concentration of 6.25 μM. Results are reported as mean and standard deviations of triplicates. These results represent one of three independent experiments.

**Figure 5:**
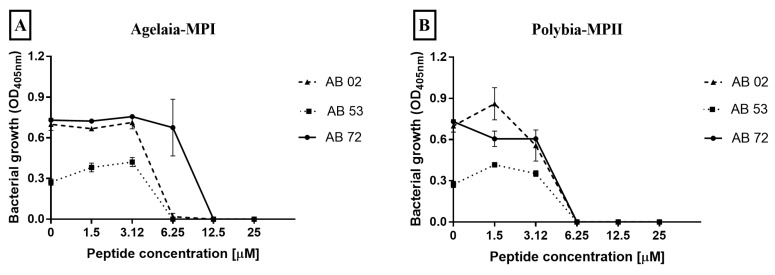
Effect of the antimicrobial peptides (**A**) Agelaia-MPI and (**B**) Polybia-MPII on *A. baumannii*-dispersed cells from biofilms on polystyrene wells. After washing the wells where biofilms had been previously formed and removing the non-adherent or poorly adhered bacteria, more culture medium was added with antimicrobial peptides and, 24 h later, bacteria present in the supernatant was measured. Results are reported as mean and standard deviations of triplicates. These results represent one of three independent experiments.

Agelaia-MPI and Polybia-MPII wasp peptides were analyzed for their ability to inhibit mature biofilm formed after 24 h of culture (Figure 4). Agelaia-MPI at 12.5 and 25 μM decreased 50% and 60% of the mature biofilm previously formed in the plates, respectively. Additionally, Agelaia-MPI and Polybia-MPII peptides were able to inhibit cells that were dispersed from the formed biofilm (Figure 5). Agelaia-MPI inhibited the dispersed cells at the minimum concentration of 12.5 μM for the AB 72 isolate and 6.25 μM for the other two (Figure 5A). Polybia-MPII inhibited the dispersed cells of all isolates at the same concentration of 6.25 μM (Figure 5B).

### 2.6. SEM Analysis of the Activity of the Agelaia-MPI and Polybia-MPII Wasp Peptides against AB 72 Isolate Biofilm adhered to the Vascular Stent

**Figure 6:**
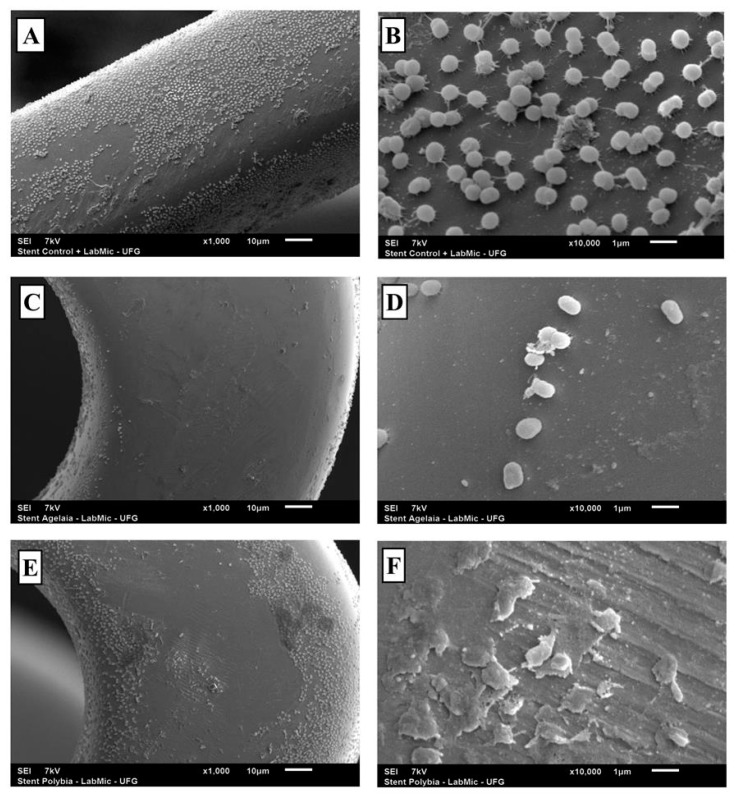
Role of the peptides Agelaia-MPI and Polybia-MPII on the inhibition of biofilm formation by *A. baumannii* on stents. (**A** and **B**) SEM evaluation of *A. baumannii* biofilms formed on stents. (**C** and **D**) Biofilm formation on stents by *A. baumannii* treated with Agelaia-MPI for 24 h. (**E** and **F**) Biofilm formation on stents by *A. baumannii* treated with Polybia-MPII for 24 h. Magnification of ×1000 (**A**, **C** and **E**); ×10,000 (**B**, **D** and **F**).

After incubating AB 72 isolate for 24 h with one fragment of vascular stent, the stents were treated with Agelaia-MPI or Polybia-MPII for 24 h (Figure 6). AMP treatment reduced the bacillary load adhered to the material and the bacteria that remained present on the stent showed morphological modifications on the bacterial surface with cellular debris accumulation (Figure 6D,F). 

### 2.7. Inhibition of Bacterial adherence on the Cobalt–hromium Stent Coated with PEG Mixed with Agelaia-MPI

**Figure 7:**
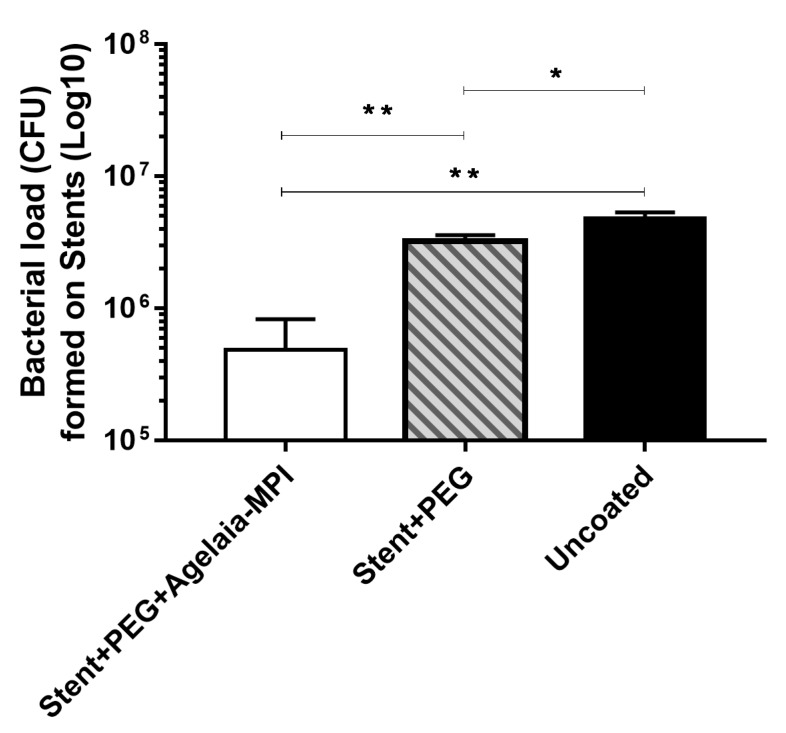
Coating of the vascular stent with Agelaia-MPI plus PEG prevented the adherence of *A. baumannii.* The vascular stent was left for 3 h in a PEG 400 solution containing 25 µM of Agelaia-MPI (stent + PEG + Agelaia−MPI) or not (stent + PEG). Coated stents were transferred to a new well containing fresh medium and then *A. baummanii* was added and incubated for 24 h. Uncoated stent was used as a control. At the end of incubation, wells were rinsed with fresh media and the stents were sonicated and the bacterial load adhered in each situation was determined. Results are reported as means and standard deviations of triplicates from one of three independent experiments. These results in all three independent experiments were similar, * *p* < 0.05 and ** *p* < 0.0001.

Stents were assembled using PEG 400 solution with Agelaia-MPI (25 µM), PEG 400 alone, or uncoated as control. Then all stents were incubated with 1.5 × 10^8^ CFU of AB 72. After 24 h, approximately 4.8 × 10^6^ CFU remained unattached to the vascular stent. Coating the stent with PEG 400 alone resulted in a slight reduction of biofilm formation (30%; ~3.25 × 10^6^ CFU). When the stent was coated with Agelaia-MPI plus PEG, a 91% reduction (~4.8 × 10^5^ CFU) was observed when compared to non-treated stents (uncoated; Figure 7).

### 2.8. Effect of Antimicrobial Peptides on Staphylococcus Biofilm Formation 

**Figure 8:**
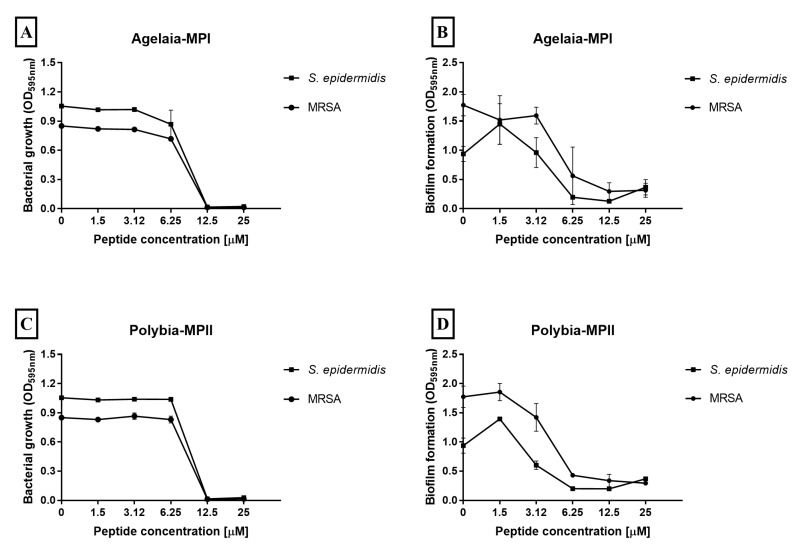
Effect of Agelaia-MPI and Polybia-MPII AMPs on the adherence and biofilm formation of *Staphylococcus* strains. (**A**) Agelaia-MPI activity against adhesion and (**B**) biofilm. Polybia-MPII activity against (**C**) adhesion and (**D**) biofilm. Results are expressed as means and standard deviations of triplicates. These results represent one of three independent experiments.

The species of *Staphylococcus* are the most common agent that causes coronary infections [4,47], thus we decided to test the microbicidal efficiency of Agelaia-MPI and Polybia-MPII peptides against *S. epidermidis* and methicillin-resistant *S. aureus* (MRSA) species. We also evaluated if the selected AMPs could avoid the formation of biofilm. Agelaia-MPI and Polybia-MPII AMPs showed similar growth inhibition at 12.5 μM for both *Staphylococcus* species (Figure 8A,C). When evaluating the biofilm formation by these bacteria, the peptides inhibited 85% of biofilm formation at 12.5 μM (Figure 8C,D). Thus, Polybia-MPII and Agelaia-MPI were microbicidal and avoided biofilm formation by *A. baumannii* and *Staphylococcus* spp. bacteria.

## 3. Discussion

Infections caused by MDR *Acinetobacter baumannii* are found in patients in hospitals due to contamination and biofilm formation of clinical materials and instruments [27]. In this work we used three *A. baumannii* clinical isolates, AB 02, AB 53, and AB 72 with resistance to different classes of antibiotics and potential biofilm formation in plates as described by Castilho et al. [43]. Here, we showed the bacterial adherence in a coronary stent with the formation of biofilm. We showed that three peptides from wasps and scorpions presented antimicrobial and antibiofilm activities. Additionally, the peptides had activity against different stages of biofilm formation: Adhesion, maturation, and dispersion. Agelia-MPI + PEG coating was used to prevent adherence of bacteria on the coronary stents and this coating reduced 90% of bacteria adhered to them. Thus, we propose that Agelaia-MPI could be an alternative therapeutic against MDR *A. baumannii* deposition and biofilm formation in clinical materials.

Two of the clinical isolates used in this study presented resistance to the beta-lactam antibiotic ampicillin and carbapenems, the aminoglycoside amikacin, and the quinolone ciprofloxacin, therefore presenting several mechanisms of drug resistance, representing a challenge to treat infections by these bacteria [43]. In recent years, there has been an increase in the number of cases of infection by MDR *A. baumannii* strains [27]. *Acinetobacter* spp. are more frequently found on inanimate objects and hands of staff in the ICU than *Staphylococcus aureus* and *Pseudomonas* spp. [27]. Another problem is the increased use of prophylactic antibiotics, which decreases the risk of infection, but increase the selection of resistant strains, such as emergent MDR *A. baumannii.* Analysis of isolates of *A. baumannii* reveal that those producing biofilms are more frequently associated with genes of antibiotic resistance compared to the weak biofilm producers [48]. In combination with different genetic profiles responsible for antimicrobial resistance, biofilm formation increases the chances of pathogen survival. Because biofilm formation may occur in medical materials, prospecting new molecules that could avoid antibiotic resistance might contribute to the treatment of such bacteria.

The strains of *A. baumannii* used in this study were capable of adherence to the cobalt–chromium structure of the vascular stent. Also, the presence of structures that resemble exopolisaccharides that support adherence to the stent were observed, which were shown be important for the beginning of biofilm formation [49]. The initial agglomeration of bacteria on surfaces might improve their resistance to desiccation and antimicrobial solutions [50]. *A. baumannii* can survive for long periods in hospital environments; many reservoirs have been identified, including mattresses, metal tables, door handles, and air vents [51]. *A. baumannii* is a cause of primarily hospital-acquired infection associated with septicemia, bacteremia, ventilator-associated pneumonia, sepsis, endocarditis, meningitis, and urinary tract infections [27]. Although contaminated stents reviewed by Bosma et al. [4] did not show the presence of *A. baumannii*, we believe that more studies should be done since *A. baumannii* can easily infect hospitalized immunosuppressed individuals and *A. baumannii* bacteremia could induce biofilm formation on the implanted stents. Very often the diagnosis of an infected stent is missed in the first phase, with a subsequent delay in definitive treatment, yet in up to 50.0% of cases it has a fatal outcome [4]. Also, wrong practices of coronary stent manipulation can lead to contamination, even when antibiotics are used preventively [52,53,54]. Our hypothesis is that there is an underestimation of cases of coronary stent infection by *A. baumannii*.

Antimicrobial peptides (AMPs) derived from wasps (Agelaia-MPI, Polybia-MPII, and Polydim-I) and scorpions (Con10 and NBDP-5.8) were tested for their ability to inhibit the growth of *A. baumannii* isolates. Among all AMPs, the Agelaia-MPI had the best MIC and MBEC values when compared to the other peptides. AMPs derived from wasp and scorpion venom have been widely tested against different microorganisms and have a microbicidal function on bacteria and fungi, besides having antiviral action [33]. Although the bactericidal activity of Agelaia-MPI against Gram-negative bacteria was not tested before, we believe that it could involve the interaction of the peptide with negatively-charged molecules on the surface of bacteria that would cause disruption of bacterial membranes [31,55]. Because AMPs acts on the lipid portion of cellular membranes, it is believed that they could avoid the development of resistance mechanisms such as those commonly induced by antibiotics, i.e., *A. baumannii* bacterial resistance mechanisms to conventional antibiotics comprises multidrug efflux pumps, aminoglycoside-modifying enzymes, selective membrane permeability, alteration of target sites, and hydrolytic enzymes like carbapenemase [56,57,58]. 

Agelaia-MPI presented the MIC of 6.25 μM for the AB 02 isolate and 3.12 μM for the AB 53 and AB 72 isolates. Such MIC variation could be due to the particular characteristics of each clinical isolate, such as membrane composition, protease secretion, etc. [59] and has been also described for antimicrobial testings [60]. The acquired antimicrobial drug resistance attributed to the biofilm formation was also observed for the isolates studied here. A reduction of 50%–60% of the bacterial load on the mature biofilms only occurred using higher AMP concentrations (12.5 and 25 μM). The *A. baumannii* reduction observed here could prevent the formation of the biofilm by killing planktonic bacteria, which reduces/eradicates mature biofilm or induce the detachment of the bacteria. In this case, Agelaia-MPI probably acts in a "classic" manner against biofilm, according to Batoni et al. [39]. When MBEC is higher than the MIC, AMP acts in a microbicidal way, preventing the biofilm by the death of the planktonic bacteria, reducing/eradicating the bacteria in the mature biofilm and finally killing those who detach from the biofilm [42]. Despite the direct correlation between AMPs concentration and bacterial death, a transient and slight bacterial growth was observed at concentrations lower than MIC (sub-MIC). Although not statistically significant, this behavior has been shown previously and explained as bacterial detachment from the biofilm that cannot be killed by sub-MIC of AMPs [61].

Agelaia-MPI and Polybia-MPII modified the bacterial surface and reduced the bacterial load on the stents (Figure 6). These peptides differ from each other by two amino acids; an alanine in Agelaia-MPI by a methionine in Polybia-MPII and an isoleucine by a valine, in the 9th and 10th position, respectively. These amino acid differences apparently did not interfere with their ability to cause membrane lesions. Polybia-MPII was shown before to present microbicidal functions against fungi (*Candida albicans* and *Cryptococcus neoformans*), *Mycobacterium abscessus* subsp. *massiliense* and *S. aureus* [31]. The ability of Polybia-MPII to avoid *A. baumannii* biofilm formation was also shown against *Staphylococcus* strains [31]. The probable mechanism of action on membrane/cell wall observed with *M. a*. *massiliense* SEM analyses were also observed for *A. baumannii* stent biofilms, thus indicating physical rather than metabolic alterations induced by the AMPs (i.e., general membrane alterations). In addition to the amino acid sequence of AMPs, some features may influence their binding to bacterial and eukaryotic membranes, i.e., hydrophobicity and the resulting charge. Increasing or diminishing hydrophobicity of AMPs has been shown to improve their bactericidal functions [62,63]. In the present work, this phenomena was not the case; Agelaia-MPI and Polybia-MPII presented similar hydrophobicity and bactericidal action, while Polydim-I, although having similar hydrophobicity, presented lower bactericidal function. Additionally, CON10 and NDBP-5.8 that presented the lowest hydrophobicity showed higher bactericidal function than Polydim-I. Thus, for the results presented here, the peptide hydrophobicity was not the only driving factor in the microbicidal activities. 

Disadvantages of the use of peptides as an antimicrobial are the production costs and their low stability in human serum, due to the action of peptidases and proteases present in the human body, especially in the liver [64]; however, there is a way to optimize the amount of peptide used and increase its stability, i.e., by using it combined with other molecules or coating medical material. Polyethylene glycol (PEG) is hydrophilic and presents low toxicity and it has been shown before to assist the slow release of AMPs such as LL37 [65]. Thus, we decided to use PEG to coat the stent with Agelia-MPI. Coating stents with Agelaia-MPI + PEG reduced 90% of the biofilm formation. Different peptides have already been used to inhibit biofilm formation; in those cases, they were adhered to silicone catheters and titanium structures [64,66]. Similar to our results, Baghery et al. immobilized AMPs with PEG and showed an improvement in the antimicrobial efficiency of the AMPs against biofilm formation [67]. Analysis comparing the immobilization of AMP with and without PEGylated spacers demonstrated that some immobile AMPs are only bactericidal when PEGylated spacer was used [65,68]. Although the works done before did not use PEG alone as control, in our case of using PEG-coated stents, the biofilm formation was reduced 30%. This fact could indicate that PEG may alter the bacteria adherence to the stent and thus avoid the complete biofilm formation, but more studies should be done to prove this fact. Thus, surface coating composed of antimicrobial peptides offers additional advantages, such as decreased potential cytotoxicity associated with higher concentrations of soluble peptides and increased peptide life [69]. 

## 4. Conclusions

In summary, this work showed two peptides, Agelaia-MPI and Polybia-MPII, derived from wasps with bactericidal activity, as well as activity against different stages of biofilm-forming by MDR *A. baumannii*. We also showed that coating cobalt–chromium vascular stents with Agelaia-MPI together with PEG 400 prevented 90% of bacterial biofilm formation. This study revealed potential applications of Agelaia-MPI and Polybia-MPII peptides derived from wasp venom as antimicrobials to treat biofilm-resistant agents such as *A. baumannii* and *Staphylococcus* spp. coated on the surfaces of implanted medical devices.

## 5. Materials and Methods 

### 5.1. Bacterial Strains and Growth Conditions

Clinical isolates of MDR *A. baumannii* described by Castilho et al. [43] identified as AB 02, AB 53, and AB 72 cryopreserved at −80 °C were reactivated in Luria Bertani (LB) agar medium (HiMedia) and grown at 37 °C for 24 h. A colony isolated from each strain was inoculated into 5 mL of LB broth medium (HiMedia, Pennsylvania, USA) until growth corresponded to 0.5 of the MacFarland scale. Some growth conditions were modified for each experiment to evaluate the different stages of biofilm formation described below.

### 5.2. Analysis of Bacterial Biofilm Formation by A. baumannii Isolates by Colorimetric Dyes in 96-Well Polystyrene Culture Plates

The estimated quantification of bacterial biofilm formation in a 96-well polystyrene culture plate was done according to methodology described by Castilho et al. [43], with minor modifications. After growth of the strains in LB broth medium to a concentration corresponding to 0.5 of MacFarland scale, the culture concentration was adjusted to 1.5 × 10^8^ CFU/mL and 30 μL of that suspension was added to 170 μL of LB broth at 1/4 of its concentration with an additional 0.2% of glucose (Ecibra) (LB¼-Glu). The bacterial culture was incubated in a 96-well plate for 24 h at a temperature of 29 °C. Bacterial growth was measured at an absorbance of 405 nm in a Thermo Scientific™ Multiskan™ FC Microplate Photometer. The supernatant was removed and the well was washed with phosphate buffered saline (PBS). The attached biofilm was stained with 0.2% (w/v) crystal violet (Vetec) and solubilized with ethanol/acetone (80/20 v/v) to quantify at 595 nm. A common laboratory *Escherichia coli* strain (XL1blue) known not to form biofilm was used as a negative control.

### 5.3. Adhesion of A. baumannii to Abiotic Surfaces

*A. baumannii* strains form bacterial biofilm on a polystyrene plate under incubation conditions (LB¼-Glu 29 °C). Biofilm formation on stents was evaluated by placing a sterile fragment of the cobalt–chromium alloy vascular stent into a well containing strain AB 72 at a concentration of 1.5 × 10^8^ CFU/mL in LB¼-Glu. As a control, a fragment of the vascular stent was incubated with culture medium alone. After 24 h of incubation, the stents were rinsed with sterile PBs and analyzed by Scanning Electron Microscopy (SEM) and CFU counting. The SEM was performed using a methodology from das Neves et al. [36], with minor modifications. The fragments were washed with PBS to remove the unbound bacteria, then fixed with Karnovsky’s solution (1% paraformaldehyde and 3% glutaraldehyde) in 0.07M cacodilide buffer (pH 7.2) for 30 min at 4 °C. The fixative solution was removed and serial dehydration was performed, followed by ethanol washes (30%, 50%, 70%, 90%, and 100%) for 10 min, followed by acetone and hexamethyldisilazane (HMDS) (v/v) for a further 5 min and ending with HMDS p.a. Lastly, they were covered with a thin layer of gold by the metallizer Denton Vacuum Desk V. The images were made using a Jeol microscope, JSM-6610, equipped with EDS (Thermo scientific NSS Spectral Imaging). The metalizations and analyses were carried out in two moments in the Multiuser Laboratories of High Resolution Microscopy of the Institute of Physics and in the Regional Center for Technological Development and Innovation and Labmic Core Facility of the Federal University of Goiás.

For CFU analyses, the rinsed stent fragments were sonicated using Ultrasonic homogenizers (SONOPULS) for 1 minute in 1 mL of cold PBS followed by serial dilution of the sonicated supernatant and plating on LB agar medium for quantification.

### 5.4. Peptides and Computational Analysis

The peptide Agelaia-MPI was derived from the Agelaia pallipes pallipes social wasp venom as described by Mendes et al. [44]. Polybia-MPII was first described by Souza et al. [45]. The AMP properties of Polydim-I were described by das Neves et al. [38]. Con10 is a peptide derived from a larger peptide sequence, described by Silva et al. [43]. This group also described the NDBP-5.8 [46]. Mass spectrometry and peptide sequence analysis were confirmed by MALDI-TOF/TOF MS (UltraFlex III, Bruker Daltonics, Germany) and LIFTTM (MS/MS) and the secondary structures of these peptides were defined [36,41,42,43]. The peptides Agelaia-MPI, Polybia-MPII, Polydim-I, Con10 and NDBP-5.8 were synthesized with terminal amidation by FastBio LTDA (Ribeirão Preto, SP Brazil) with >95% purity.

### 5.5. Determination of Minimum Inhibitory Concentration (MIC)

The minimum inhibitory concentration was determined by broth microdilution according to Wiegand et al. [70]. The AB 02, AB 52 and AB 72 isolates were grown in LB broth medium for 8 h at 37 °C until they reached a growth turbidity corresponding to 0.5 of the McFarland scale. Thirty microliters of each culture were added in the wells of polystyrene plates containing 170 µL of culture medium with different concentrations of the antimicrobial peptides. Agelaia-MPI, Polybia-MPII, Polydim-I, Con10 and NDBP 5.8 antimicrobial peptides were serially diluted from 25 to 1.56 μM. The plate containing different concentrations of individual peptides with the bacterial strains were incubated for 24 h at 37 °C, after which time the plates were read at the optical density of 405 nm. As positive control, bacterial strains without peptides were grown in the same conditions, and as negative control medium alone was used.

### 5.6. Determination of the Minimal Inhibitory Concentration for Biofilm Formation

In order to determine if individual peptides could avoid biofilm formation, the minimal biofilm eradication concentration (MBEC) determination assay was performed using 96-well polyethylene plates according to Feng et al. [71] methodology with minor modifications. Initially, *A. baumannii* strains (AB 02, AB 53, and AB 72) were grown for 6 h in LB broth until reaching an optimum growth of 1.0 in the optical density of 600 nm and then the culture concentration was adjusted to 1.5 × 10^8^ CFU/mL and 30 μL of the culture was added to the wells containing 170 μL de LB ¼ + 0.2% glucose containing serially diluted peptides ranging from 25 to 1.56 μM. Cells were incubated at 29 °C for 24 h to let biofilms be formed. The supernatant was removed and the wells were washed with 200 μL of PBS twice to remove the non-adherent and weakly adherent cells, maintaining only the mature biofilm formed on the plate. Biofilm quantification was measured by staining attached cells with 0.2% (w/v) violet crystal staining solubilized with ethanol/acetone (80/20 v/v) and quantified at 595 nm. The biofilms formed in cultures with different peptide concentrations were compared with the biofilms formed in cultures of *A. baumannii* strains without peptides. Analyses were performed in triplicate and three independent experiments were performed for each of these assays.

### 5.7. Analysis of the Removal of Mature Bacterial Biofilm by Antimicrobial Peptides

The release of substrate produced by the bacterium itself conferred resistance to different antimicrobials. Knowing this, after the adhesion and structuring of the bacterial biofilms on the exogenous surface, we analyzed the potency of the Agelaia-MPI and Polybia-MPII peptides in removing the mature biofilm of *A. baumannii* strains. The experiment followed the methodology similar to the previous ones, except that the peptide was added after the formation of the plaque biofilm. The biofilm was quantified by the absorption of violet crystal after 24 h of contact with the antimicrobial peptides at the usual concentrations.

### 5.8. Inhibition of the Dispersion of Bacteria from the Biofilm by the Antimicrobial Peptides

The third stage of the biofilm formation cycle is the dispersion of bacterial cells from the adhered material to the medium. To investigate whether the peptides were able to inhibit this stage, the bacterial isolates were cultivated until they formed biofilms (LB¼-Glu), following the methodology described above. Biofilm-containing wells were washed 3 times with PBS to remove non-adherent and weakly adherent cells. The peptides were serially diluted and added to the biofilms at final concentrations ranging from 25 to 1.56 μM and the plate was incubated again for 24 h. Dispersion inhibition was measured by quantifying the bacteria that dispersed from the biofilm and was detected in the culture medium by determining the OD at 405 nm of the supernatants. Controls consisted of media-only (negative) and bacteria without peptides (positive).

### 5.9. Stent Biofilm Formation Inhibition by Agelaia-MPI Complexed with Polyethylene Glycol (PEG) 

Sterile coronary stent fragments were placed in contact with a PEG 400 solution containing 25 μM Agelaia-MPI for 3 h. At the end of that period, the stent fragments were removed from solution and placed in tubes containing LB¼ + GLU and AB 72 isolate at a concentration of 1.5 × 10^8^ CFU/mL and incubated at 29 °C for 24 h. After this period, the stents were washed with PBS. For CFU analyses, the stent fragments were sonicated for 1 minute in 1 mL of cold PBS, dilutions were made and plated on LB agar medium for quantification. As controls, the amount of bacteria adhered to stents without any treatment or treated with PEG only were evaluated.

### 5.10. Analysis of Bacterial Biofilm Formation of Staphylococcus Strains by Colorimetry

To determine the formation of the biofilm of *Staphylococcus* spp., the methodology according to Kwasny and Opperman (2010) with modifications was used. *Staphylococcus epidermidis* and methicillin-resistant *Staphylococcus aureus* (MRSA) cryopreserved strains at −80 °C were reactivated in solid medium to obtain an isolated colony, which was inoculated in LB medium broth and cultured at 37 °C until they reached a concentration corresponding to 0.5 turbidity of the MacFarland scale [72]. At that concentration, 30 μL of the bacterial culture was added to wells containing 170 μL of tryptic soy broth (HiMedia) broth with 1% glucose. The plate was incubated at 37 °C for 24 h. After this period the bacterial growth was quantified by optical density reading at 600 nm and the formation of the biofilm was determined by the colorimetric method similar to that used for *Acinetobacter*. The biofilm inhibition test also followed the standard for the previous tests, with modifications in the culture medium used to increase the bacterial biofilm production by *Staphylococcus* strains.

### 5.11. Statistical Analysis

Two-way ANOVA with Tukey’s post-test multiple comparisons test was used to determine the difference in biofilm production between strains and the difference between inhibitions of the different concentrations of the peptides used. All data are presented as mean ± SD and *p* < 0.05 indicates a significant difference between groups. Data were tabulated using Excel software and the mean and standard deviation values were calculated. To evaluate the statistical differences between the groups, the software GraphPad Prism 6.0 was used (GraphPad Software, Inc., San Diego, CA, USA).

## Figures and Tables

**Table 1:** Description of the sequence, source, and publication of the antimicrobial peptides used in this study.

Peptide	Sequence	Venom source	Hydrophobicity	First Description
Agelaia-MPI	INWLKLGKAIIDAL	*Agelaia pallipes pallipes*	0.781	[44]
Polybia-MPII	INWLKLGKMVIDAL	*Pseudopolybia vespiceps testacea*	0.740	[45]
Polydim-I	AVAGEKLWLLPHLLKMLLTPTP	*Polybia dimorpha*	0.791	[38]
Con10	FWSFLVKAASKILPSLIGGGDDNKSSS	*Opisthacanthus cayaporum*	0.435	[46]
NDBP-5.8	GILGKIWEGVKSLI	*Opisthacanthus cayaporum*	0.686	[46]

**Table 2:** Minimal inhibitory concentration of antimicrobial peptides against the formation of *A. baumannii* biofilm on polystyrene plates.

Peptide	Isolate AB 02 ^1^	Isolate AB 53	Isolate AB 72
Agelaia-MPI	25	6.25	12.5
Polybia-MPII	25	12.5	25
Polydim-I	>25	>25	>25
Con 10	25	12.5	12.5
NDBP 5.8	>25	>25	>25

^1^ Values are presented as concentration in µM that inhibited biofilm formation after violet crystal staining of the biofilm adhered to polystyrene plate by reading OD in the range of 595 nm.

## References

[1] Minardi D., Ghiselli R., Cirioni O., Giacometti A., Kamysz W., Orlando F., Silvestri C., Parri G., Kamysz E., Scalise G. (2007). The antimicrobial peptide Tachyplesin III coated alone and in combination with intraperitoneal piperacillin-tazobactam prevents ureteral stent Pseudomonas infection in a rat subcutaneous pouch model. Peptides.

[2] De Breij A., Riool M., Kwakman P.H.S., de Boer L., Cordfunke R.A., Drijfhout J.W., Cohen O., Emanuel N., Zaat S.A.J., Nibbering P.H. (2016). Prevention of Staphylococcus aureus biomaterial-associated infections using a polymer-lipid coating containing the antimicrobial peptide OP-145. J. Control. Release.

[3] Arciola C.R., Campoccia D., Montanaro L. (2018). Implant infections: Adhesion, biofilm formation and immune evasion. Nat. Rev. Microbiol..

[4] Bosman W.M.P.F., Borger van der Burg B.L.S., Schuttevaer H.M., Thoma S., Hedeman Joosten P.P. (2014). Infections of intravascular bare metal stents: A case report and review of literature. Eur. J. Vasc. Endovasc. Surg..

[5] Dalal J.J., Digrajkar A., Hastak M., Mulay A., Lad V., Wani S. (2017). Coronary stent infection—A grave, avoidable complication. IHJ Cardiovasc. Case Rep..

[6] Reddy K.V.C., Sanzgiri P., Thanki F., Suratkal V. (2018). Coronary stent infection: Interesting cases with varied presentation. J. Cardiol. Cases.

[7] Elieson M., Mixon T., Carpenter J. (2012). Coronary stent infections. Tex. Heart Inst. J..

[8] Whitcher G.H., Bertges D.J., Shukla M. (2018). Peripheral vascular stent infection: Case report and review of literature. Ann. Vasc. Surg..

[9] Desai J.A., Husain S.F., Islam O., Jin A.Y. (2010). Carotid artery stent infection with Streptococcus agalactiae. Neurology.

[10] Sekhar S., Vupputuri A., Nair R.C., Palaniswamy S.S., Natarajan K.U. (2016). Coronary stent infection successfully diagnosed using 18F-flurodeoxyglucose positron emission tomography computed tomography. Can. J. Cardiol..

[11] Sudhakar B.G.K. (2018). Pseudomonas aeruginosa septicemia resulting in coronary stent infection and coronary artery aneurysm and acute infective endocarditis of mitral valve causing severe mitral regurgitation—A case report. IHJ Cardiovasc. Case Rep..

[12] Raychaudhuri R., Yu W., Hatanpaa K., Cavuoti D., Pride G.L., White J. (2009). Basilar artery dissection treated by Neuroform stenting: Fungal stent infection. Surg. Neurol..

[13] Soman R., Gupta N., Suthar M., Sunavala A., Shetty A., Rodrigues C. (2015). Intravascular stent-related endocarditis due to rapidly growing mycobacteria: A new problem in the developing world. J. Assoc. Phys. India.

[14] Wu X., Yin T., Tian J., Tang C., Huang J., Zhao Y., Zhang X., Deng X., Fan Y., Yu D. (2015). Distinctive effects of CD34- and CD133-specific antibody-coated stents on re-endothelialization and in-stent restenosis at the early phase of vascular injury. Regen. Biomater..

[15] DeCunha J., Janicki C., Enger S.A. (2017). A retrospective analysis of catheter-based sources in intravascular brachytherapy. Brachytherapy.

[16] Abhyankar A.D., Thakkar A.S. (2012). In vivo assessment of stent recoil of biodegradable polymer-coated cobalt–chromium sirolimus-eluting coronary stent system. Indian Heart J..

[17] Buccheri D., Orrego P.S., Cortese B. (2015). Drug-eluting stent treatment of left main coronary artery disease: The case for a sirolimus-eluting, autoexpandable alternative. An optical coherence tomography analysis. Int. J. Cardiol..

[18] Ito S., Saeki T. (2015). Coronary angioscopic imaging of in-stent restenosis after biolimus-eluting coronary stent implantation. J. Cardiol. Cases.

[19] Li C.H., Gao B.L., Wang J.W., Liu J.F., Li H., Yang S.T., Ren C.F. (2018). Endovascular stent deployment in the management of lesions related to internal carotid artery redundancy. World Neurosurg..

[20] Omar A., Pendyala L.K., Ormiston J.A., Waksman R. (2016). Review: Stent fracture in the drug-eluting stent era. Cardiovasc. Revasc. Med..

[21] Zumstein V., Betschart P., Albrich W.C., Buhmann M.T., Ren Q., Schmid H.P., Abt D. (2017). Biofilm formation on ureteral stents—Incidence, clinical impact and prevention. Swiss Med. Wkly..

[22] Garrett T.R., Bhakoo M., Zhang Z. (2008). Bacterial adhesion and biofilms on surfaces. Prog. Nat. Sci..

[23] Elbadawi A., Saad M., Elgendy I.Y., Zafar A., Chow M.-Y. (2017). Multiple myocardial abscesses secondary to late stent infection. Cardiovasc. Pathol..

[24] Babapour E., Haddadi A., Mirnejad R., Angaji S.-A., Amirmozafari N. (2016). Biofilm formation in clinical isolates of nosocomial Acinetobacter baumannii and its relationship with multidrug resistance. Asian Pac. J. Trop. Biomed..

[25] Longo F., Vuotto C., Donelli G. (2014). Biofilm formation in Acinetobacter baumannii. New Microbiol..

[26] Liu C.P., Shih S.C., Wang N.Y., Wu A.Y., Sun F.J., Chow S.F., Chen T.L., Yan T.R. (2016). Risk factors of mortality in patients with carbapenem-resistant Acinetobacter baumannii bacteremia. J. Microbiol. Immunol. Infect..

[27] Almasaudi S.B. (2018). *Acinetobacter* spp. as nosocomial pathogens: Epidemiology and resistance features. Saudi J. Biol. Sci..

[28] Deiham B., Douraghi M., Adibhesami H., Yaseri M., Rahbar M. (2017). Screening of mutator phenotype in clinical strains of Acinetobacter baumannii. Microb. Pathog..

[29] Djeribi R., Bouchloukh W., Jouenne T., Menaa B. (2012). Characterization of bacterial biofilms formed on urinary catheters. Am. J. Infect. Control.

[30] Cobrado L., Silva-Dias A., Azevedo M.M., Pina-Vaz C., Rodrigues A.G. (2013). In vivo antibiofilm effect of cerium, chitosan and hamamelitannin against usual agents of catheter-related bloodstream infections. J. Antimicrob. Chemother..

[31] Silva J.C., Neto L.M., Neves R.C., Gonçalves J.C., Trentini M.M., Mucury-Filho R., Smidt K.S., Fensterseifer I.C., Silva O.N., Lima L.D. (2017). Evaluation of the antimicrobial activity of the mastoparan Polybia-MPII isolated from venom of the social wasp Pseudopolybia vespiceps testacea (Vespidae, Hymenoptera). Int. J. Antimicrob. Agents.

[32] Pluzhnikov K.A., Kozlov S.A., Vassilevski A.A., Vorontsova O.V., Feofanov A.V., Grishin E.V. (2014). Linear antimicrobial peptides from Ectatomma quadridens ant venom. Biochimie.

[33] Perumal Samy R., Stiles B.G., Franco O.L., Sethi G., Lim L.H.K. (2017). Animal venoms as antimicrobial agents. Biochem. Pharmacol..

[34] Garcia F., Villegas E., Espino-Solis G.P., Rodriguez A., Paniagua-Solis J.F., Sandoval-Lopez G., Possani L.D., Corzo G. (2013). Antimicrobial peptides from arachnid venoms and their microbicidal activity in the presence of commercial antibiotics. J. Antibiot..

[35] Harrison P.L., Abdel-Rahman M.A., Miller K., Strong P.N. (2014). Antimicrobial peptides from scorpion venoms. Toxicon.

[36] Souza B.M.D., Cabrera M.P.D.S., Gomes P.C., Dias N.B., Stabeli R.G., Leite N.B., Neto J.R., Palma M.S. (2015). Structure-activity relationship of mastoparan analogs: Effects of the number and positioning of Lys residues on secondary structure, interaction with membrane-mimetic systems and biological activity. Peptides.

[37] Wang K., Yan J., Dang W., Xie J., Yan B., Yan W., Sun M., Zhang B., Ma M., Zhao Y. (2014). Dual antifungal properties of cationic antimicrobial peptides polybia-MPI: Membrane integrity disruption and inhibition of biofilm formation. Peptides.

[38] Das Neves R.C., Trentini M.M., de Castro e Silva J., Simon K.S., Bocca A.L., Silva L.P., Mortari M.R., Kipnis A., Junqueira-Kipnis A.P. (2016). Antimycobacterial activity of a new peptide polydim-I isolated from neotropical social Wasp Polybia dimorpha. PLoS ONE.

[39] Moerman L., Bosteels S., Noppe W., Willems J., Clynen E., Schoofs L., Thevissen K., Tytgat J., Van Eldere J., Van Der Walt J. (2002). Antibacterial and antifungal properties of ??-helical, cationic peptides in the venom of scorpions from southern Africa. Eur. J. Biochem..

[40] O’Brien-Simpson N.M., Hoffmann R., Chia C.S.B.S.B., Wade J.D.D., Brien-Simpson N.M.O., Hoffmann R., Chia C.S.B.S.B., Wade J.D.D. (2018). Antimicrobial and Anticancer Peptides.

[41] Brogden K.A. (2005). Antimicrobial peptides: Pore formers or metabolic inhibitors in bacteria?. Nat. Rev. Microbiol..

[42] Batoni G., Maisetta G., Esin S. (2016). Antimicrobial peptides and their interaction with biofilms of medically relevant bacteria. Biochim. Biophys. Acta Biomembr..

[43] Castilho S.R.A., Godoy C.S.D.M., Guilarde A.O., Cardoso J.L., André M.C.P., Junqueira-Kipnis A.P., Kipnis A. (2017). Acinetobacter baumannii strains isolated from patients in intensive care units in Goiânia, Brazil: Molecular and drug susceptibility profiles. PLoS ONE.

[44] Mendes M.A., De Souza B.M., Marques M.R., Palma M.S. (2004). Structural and biological characterization of two novel peptides from the venom of the neotropical social wasp Agelaia pallipes pallipes. Toxicon.

[45] De Souza B.M., Marques M.R., Tomazela D.M., Eberlin M.N., Mendes M.A., Palma M.S. (2004). Mass spectrometric characterization of two novel inflammatory peptides from the venom of the social waspPolybia paulista. Rapid Commun. Mass Spectrom..

[46] Silva É.C.N., Camargos T.S., Maranhão A.Q., Silva-Pereira I., Silva L.P., Possani L.D., Schwartz E.F. (2009). Cloning and characterization of cDNA sequences encoding for new venom peptides of the Brazilian scorpion Opisthacanthus cayaporum. Toxicon.

[47] Kaufmann B.A., Kaiser C., Pfisterer M.E., Bonetti P.O. (2005). Coronary stent infection: A rare but severe complication of percutaneous coronary intervention. Swiss Med. Wkly..

[48] Sanchez C.J., Mende K., Beckius M.L., Akers K.S., Romano D.R., Wenke J.C., Murray C.K. (2013). Biofilm formation by clinical isolates and the implications in chronic infections. BMC Infect. Dis..

[49] Espinal P., Martí S., Vila J. (2012). Effect of biofilm formation on the survival of Acinetobacter baumannii on dry surfaces. J. Hosp. Infect..

[50] Bravo Z., Chapartegui-González I., Lázaro-Díez M., Ramos-Vivas J. (2018). Acinetobacter pittii biofilm formation on inanimate surfaces after long-term desiccation. J. Hosp. Infect..

[51] Munier A.-L., Biard L., Rousseau C., Legrand M., Lafaurie M., Lomont A., Donay J.-L., de Beaugrenier E., Flicoteaux R., Mebazaa A. (2017). Incidence, risk factors, and outcome of multidrug-resistant Acinetobacter baumannii acquisition during an outbreak in a burns unit. J. Hosp. Infect..

[52] Furtado A.D., Bhat S.P.S., Peer S.M., Chikkatur R. (2011). Infected pseudoaneurysm involving a drug-eluting stent. Interact. Cardiovasc. Thorac. Surg..

[53] Venkatesan A.M., Kundu S., Sacks D., Wallace M.J., Wojak J.C., Rose S.C., Clark T.W.I., D’Othee B.J., Itkin M., Jones R.S. (2010). Practice guideline for adult antibiotic prophylaxis during vascular and interventional radiology procedures. J. Vasc. Interv. Radiol..

[54] Schoenkerman A.B., Lundstrom R.J. (2009). Coronary stent infections: A case series. Catheter. Cardiovasc. Interv..

[55] Vila-Farrés X., López-Rojas R., Pachón-Ibáñez M.E., Teixidó M., Pachón J., Vila J., Giralt E. (2015). Sequence-activity relationship, and mechanism of action of mastoparan analogues against extended-drug resistant Acinetobacter baumannii. Eur. J. Med. Chem..

[56] Huang Y., Wiradharma N., Xu K., Ji Z., Bi S., Li L., Yang Y.Y., Fan W. (2012). Cationic amphiphilic alpha-helical peptides for the treatment of carbapenem-resistant Acinetobacter baumannii infection. Biomaterials.

[57] Lin C.H., Lee M.C., Tzen J.T.C., Lee H.M., Chang S.M., Tu W.C., Lin C.F. (2017). Efficacy of mastoparan-AF alone and in combination with clinically used antibiotics on nosocomial multidrug-resistant Acinetobacter baumannii. Saudi J. Biol. Sci..

[58] Lin M.-F. (2014). Antimicrobial resistance in *Acinetobacter baumannii*: From bench to bedside. World J. Clin. Cases.

[59] Wang Y.C., Kuo S.C., Yang Y.S., Lee Y.T., Chiu C.H., Chuang M.F., Lin J.C., Chang F.Y., Chen T.L. (2016). Individual or combined effects of meropenem, imipenem, sulbactam, colistin, and tigecycline on biofilm-embedded Acinetobacter baumannii and biofilm architecture. Antimicrob. Agents Chemother..

[60] Andreassen S., Zalounina A., Paul M., Sanden L., Leibovici L. (2015). Interpretative reading of the antibiogram—Semi-naïve Bayesian approach. Artif. Intell. Med..

[61] Ammann C.G., Neuhauser D., Eberl C., Nogler M., Coraça-Huber D. (2018). Tolerance towards gentamicin is a function of nutrient concentration in biofilms of patient-isolated Staphylococcus epidermidis. Folia Microbiol..

[62] Saint Jean K.D., Henderson K.D., Chrom C.L., Abiuso L.E., Renn L.M., Caputo G.A. (2018). Effects of hydrophobic amino acid substitutions on antimicrobial peptide behavior. Probiotics Antimicrob. Proteins.

[63] Andreev K., Martynowycz M.W., Huang M.L., Kuzmenko I., Bu W., Kirshenbaum K., Gidalevitz D. (2018). Hydrophobic interactions modulate antimicrobial peptoid selectivity towards anionic lipid membranes. Biochim. Biophys. Acta Biomembr..

[64] Mishra B., Lushnikova T., Golla R.M., Wang X., Wang G. (2017). Design and surface immobilization of short anti-biofilm peptides. Acta Biomater..

[65] Gabriel M., Nazmi K., Veerman E.C., Amerongen A.V.N., Zentner A. (2006). Preparation of LL-37-grafted titanium surfaces with bactericidal activity. Bioconjug. Chem..

[66] Mishra B., Basu A., Chua R.R.Y., Saravanan R., Tambyah P.A., Ho B., Chang M.W., Leong S.S.J. (2014). Site specific immobilization of a potent antimicrobial peptide onto silicone catheters: Evaluation against urinary tract infection pathogens. J. Mater. Chem. B.

[67] Bagheri M., Beyermann M., Dathe M. (2009). Immobilization reduces the activity of surface-bound cationic antimicrobial peptides with no influence upon the activity spectrum. Antimicrob. Agents Chemother..

[68] Costa F., Carvalho I.F., Montelaro R.C., Gomes P., Martins M.C.L. (2011). Covalent immobilization of antimicrobial peptides (AMPs) onto biomaterial surfaces. Acta Biomater..

[69] Singha P., Locklin J., Handa H. (2017). A review of the recent advances in antimicrobial coatings for urinary catheters. Acta Biomater..

[70] Wiegand I., Hilpert K., Hancock R.E.W. (2008). Agar and broth dilution methods to determine the minimal inhibitory concentration (MIC) of antimicrobial substances. Nat. Protoc..

[71] Feng X., Sambanthamoorthy K., Palys T., Paranavitana C. (2013). The human antimicrobial peptide LL-37 and its fragments possess both antimicrobial and antibiofilm activities against multidrug-resistant Acinetobacter baumannii. Peptides.

[72] Kwasny S.M., Opperman T.J. (2010). Static biofilm cultures of gram positive pathogens grown in a microtiter format used for anti-biofilm drug discovery. Curr. Protoc. Pharmacol..

